# Resveratrol induces cell cycle arrest and apoptosis with docetaxel in prostate cancer cells via a p53/ p21^WAF1/CIP1^ and p27^KIP1^ pathway

**DOI:** 10.18632/oncotarget.15303

**Published:** 2017-02-04

**Authors:** Santosh Kumar Singh, Saswati Banerjee, Edward P. Acosta, James W. Lillard, Rajesh Singh

**Affiliations:** ^1^ Department of Microbiology, Biochemistry and Immunology, Morehouse School of Medicine, Atlanta, GA, USA; ^2^ Department of Pharmacology & Toxicology, University of Alabama at Birmingham, Birmingham, AL, USA

**Keywords:** cell cycle, resveratrol, docetaxel, p21, p53

## Abstract

Resveratrol (RES) is the most effective natural products used for the treatment of a variety of cancers. In this study, we tested the effect of RES in enhancing the efficacy of docetaxel (DTX) treatment in prostate cancer (PCa) cells. The C4-2B and DU-145 cell lines were treated with RES, DTX and combination followed by evaluating the apoptosis and cell cycle progression. The combined drug treatment up-regulates the pro-apoptotic genes (*BAX, BID*, and *BAK*), cleaved *PARP* and down regulates the anti-apoptotic genes (*MCL-1, BCL-2, BCL-XL*) promoting apoptosis. In C4-2B cells the combination up regulated the expression of *p53*, and cell cycle inhibitors (p21^WAF1/CIP1^, p27^KIP^), which, in turn, inhibited the expression of CDK4, cyclin D1, cyclin E1 and induced hypo-phosphorylation of Rb thus blocking the transition of cells in the G_0_/G_1_ to S phase. In contrast, the synergistic effect was not profound in DU145 due to its lesser sensitivity to DTX. The suppression of cyclin B1 and CDK1 expression in both cell lines inhibits the further progression of cells in G_2_/M phase. The current study demonstrates that combination treatment blocks the cell cycle arrest by modulation of key regulators and promotes apoptosis via *p53* dependent and independent mechanism in PCa.

## INTRODUCTION

Prostate cancer (PCa) is one of the most leading causes of cancer-related death worldwide. In 2016, approximately 180,890 cases of PCa were newly diagnosed and 26,120 deaths have been estimated in the United States [1]. Chemotherapeutic drug docetaxel (DTX) is a well-known microtubule-stabilizing agent and commonly used during the treatment of hormone-refractory PCa patients [2]. Unfortunately, long-term treatment of DTX acquired resistance in PCa is due to microtubule mutations and activation of drug efflux pumps [3, 4]. There are many apoptosis regulators, genetic and epigenetic factors involved in the initiation, progression, and metastasis of human PCa malignancy [5].

Resveratrol (RES) (3,4,5-trihydroxystilbene), a polyphenolic phytoalexin is commonly available in natural compounds such as grapes, berries, peanuts, and soybeans. RES is a promising chemopreventive molecule because of its antioxidant, anti-inflammatory and growth inhibitory effects, and is also known to be advantageous in cardiovascular diseases [6–8]. It has been reported to modulate several apoptotic (PARP, Cleaved Caspases), pro-apoptotic (BAX, BID, BNIP3, BAK) and anti-apoptotic (MCL-1, BCL-XL, BCL-2) proteins, but very few reports are available regarding the underlying mechanisms defining the interactions of those key players involved in the cell cycle progression and cell death. Previous studies showed that RES induced anti-proliferation/apoptosis in various cancers, such as prostate, breast, colon, gastric and melanoma [5, 9–11], and was also found to be involved in the inhibition of ABC transporters, and regulation of several pathways such as *PTEN/AKT* [5].

The relationship between cell cycle and apoptosis was evident in the role of *p53*, which is a well-known tumor suppressor gene that induces the target genes p21^WAF1/CIP1^ and *BAX* for cell cycle arrest and cell death [12]. Cell proliferation is mediated by several signaling molecules and checkpoints (CDKs) that regulate cell division. The progression through the cell cycle is positively regulated by cyclins (D and E)/cyclin-dependent kinases (CDK4, CDK6, and CDK2) complex, which phosphorylates retinoblastoma tumor suppressor (pRb) protein for the transition of cells from G_1_ to S phase, with the release of the E2F transcription factors. However, the kinase inhibitor proteins p21^WAF1/CIP1^, p27^KIP1^ and p57 ^KIP2^ binds to cyclin D/ CDKs (4 or 6) complex or cyclin E/ CDK2 complex and block G_1_/S transition. Other protein families (e.g. INK4) were also reported to bind to the cyclin/CDKs involved and inhibiting the progression of G_1_ phase. Besides these, CDK1/cyclin (A or B) complex mediates the role of cell cycle progression into G_2_ and M phase [13].

Previous studies suggested that RES inhibited the cell proliferation by interfering with the several transcriptional factors. Another study on RES reveals that lack of apoptosis is regulated by the cell cycle inhibitors (p21^WAF1/CIP1^, p27^KIP1^ and p53), cyclin-dependent kinase (CDKs) and other transcription factors [7, 14]. Several reports have demonstrated that RES interfere in the cell cycle progression by blocking the G_1_/S or G_2_/M phase on different cancers [15–17]. The p21^WAF1/CIP1^ and p27^KIP1^ expressions are induced by *p53*, which is a well-known inhibitor of CDKs family proteins. Previous reports have also shown that p21^WAF1/CIP1^ plays an important role in apoptosis by inducing pro-apoptotic and inhibiting the anti-apoptotic proteins, and it also lead to cell cycle arrest in androgen-dependent and independent PCa cell lines. Moreover, less expression of p27^KIP1^ was reported to be associated with the aggressiveness of PCa [18, 19]. The androgen receptor was reported to regulate the G_1_/S transition, CDKs activity, and *pRb* gene, which controls the androgen-dependent cell proliferation in PCa [20]. Therefore, triggering the pathways for apoptosis and blocking the cell cycle progression could be the new approach for the treatment of PCa.

In the current study, we used resveratrol (RES), a natural compound with chemopreventive potential, to test its ability to enhance the effectiveness of docetaxel (DTX), as well as, to explore the property of the combined drug treatment (RES+DTX) in the cell cycle modulation of androgen independent (AI) PCa cell lines.

## RESULTS

### Effect of resveratrol and docetaxel alone or in combination on viability, cytotoxicity, and apoptosis of PCa cells

**Figure 1:**
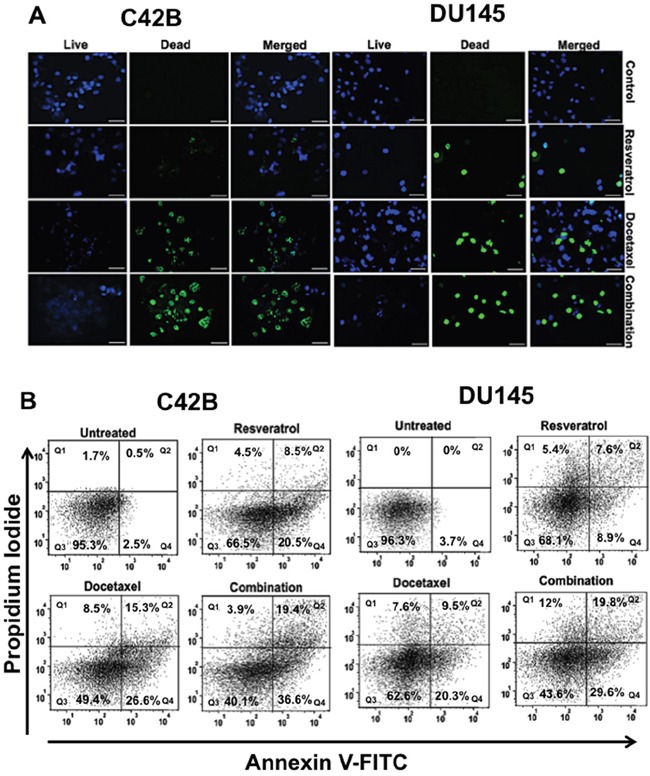
Effect of resveratrol and docetaxel alone or combinations on viability, cytotoxicity, and apoptosis in PCa cells **A**. C4-2B cells were treated with different doses of RES (47μM), DTX (10nM), and RES+DTX (20μM, 10nM) for 48 h, and were processed for immunofluorescent staining. It showed abundant expression of live nuclei in control compare to any treatment group, whereas the number of dead nuclei was more within the cells treated with the combination of both drugs. Similarly; in DU145 cells, most of the dead nuclei were found in the treatment of RES+DTX (22μM, 10nM) compared to the control (DMSO +Cells), (RES (35μM) or DTX (31nM) alone. Nuclei of live cells were detected by DAPI filter, dead cells were detected by GFP filter, represented as blue and green colors respectively. Scale bar represents 100μm. **B**. PCa cells were treated with resveratrol, docetaxel alone or in combination (RES+DTX) at 48 h, and apoptosis was evaluated by Annexin V-FITC and PI staining followed by flow cytometry. The bold number in quadrant indicates the percentage of early Q4: (Annexin(+)/(PI(-), late Q2: (Annexin(+)/(PI(+), necrotic Q1: (Annexin(-)/(PI(+), and viable Q3: Annexin(-)/(PI(-) cells respectively. The maximum number of apoptotic cells was found in the right lower (early) or upper (late) quadrant, respectively for a combination of drug (RES+DTX) for both PCa cell lines. Data are representative mean of three independent experiments ± s.d.

The cytotoxic effect of resveratrol (RES) at 24 and 72 h was not effectve, but at 48 h, resveratrol-induced apoptosis was prominent in a dose-dependent manner either alone or in combination with docetaxel (DTX). The viability assay determined the optimal IC_50_ values of RES, DTX and combination of drugs for apoptosis in C4-2B and DU145 cells. In order to establish whether or not the collective effects of RES and DTX were synergistic, the combination index (CI) was calculated according to the Chou and Talalay median effect principle [21]. Drugs were applied to PCa cells at concentration relative to their respective IC_50_ values keeping the ratio of one drug to the other constant. The relative growth rates were calculated in comparison with PCa cells in the absence of any cytotoxic drugs. The C4-2B cells had IC_50_ values, 47μM (RES), 10nM (DTX) and DU145 cells had 35μM (RES), 31nM (DTX). The combination Index was found to be 0.56 (CI= 0.56) in C4-2B cells treated with 20μM RES and 10nM DTX and 0.87 (CI=0.87) for DU145 cells treated with 22μM RES and 10nM DTX after 48h of treatment. This data suggests that the synergistic effect of RES+DTX was more efficient in C4-2B cell line compared to DU145 cell line after 48 h of treatment. To determine the viability, cells were stained, which gives the blue and green color of live and dead nuclei, respectively. These immunofluorescent images (Figure 1A) further confirm that in both C4-2B and DU145 cells, dead nuclei were found within the cells treated by the combination of RES+DTX compared to RES or DTX alone.

To evaluate the synergistic effect of resveratrol and docetaxel on apoptosis, PCa cell lines were treated with 47μM (RES), 10nM (DTX), 20μM +10nM (RES+DTX) for C4-2B and 35μM (RES), 31nM (DTX), 22μM+10nM (RES+DTX) for DU145 for 48 h and upon treatment, cells were stained with Annexin V-FITC and PI and analyzed by FlowJo software version 10.2. In the Figure 1B, data were shown in quadrant Q1, Q2, Q3 and Q4, which represent necrotic [Annexin (-)/ PI (+)], late apoptotic [Annexin (+)/ PI (+)], viable [Annexin (-)/PI (-)] and early apoptotic [Annexin (+)/PI (-)] cells respectively. In C4-2B cells, apoptosis induced by a combination of drugs was significantly higher (early 36.6% and 19.4% late) compared to the cell treated with DTX (early 26.6% and late 15.3%) and RES (early20.5% and late 8.5%) only. However, the combined treatment of (RES+DTX) in DU145 cells showed early, and late apoptosis, which is 29.6% and 19.8%, respectively, compared to DTX (early 20.3% and late 9.5%) and RES (early 8.9% and late 7.6%) alone. These results signify the role of RES in combination with DTX, which induced the apoptosis in both PCa cells.

### Resveratrol-induced the modulation of pro- and anti-apoptotic protein in PCa cells

**Figure 2:**
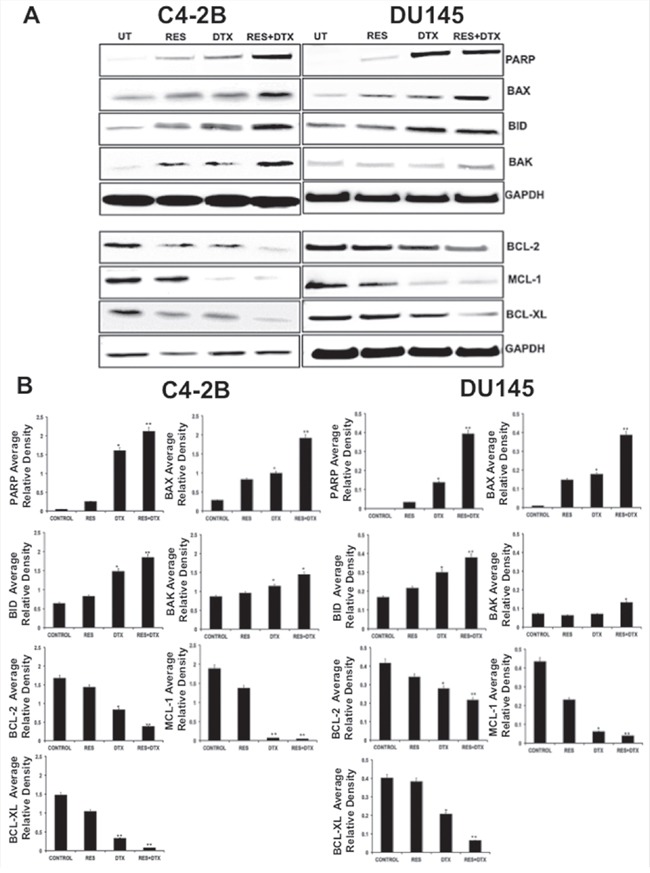
Detection of pro-apoptotic, anti-apoptotic and apoptotic protein in C4-2B and DU145 PCa cells by immunoblotting **A**. Expression of pro- and anti- apoptotic proteins in C4-2B cells after treatment with RES (47μM), DTX (10nM) and combination (RES+DTX (20μM, 10nM) after 48 h, represents up-regulation of pro-apoptotic proteins (BAX, BID and BAK), cleaved PARP, and down- regulation of anti-apoptotic markers (BCL-2, BCL-XL, and MCL-1). Similar expression of pro- and anti-apoptotic proteins were found in DU145 cells after treatment with RES (35μM); DTX (31nM) and combination (RES+DTX (22μM, 10nM) for 48h treatment. The representative immunoblots are shown with their respective cell lines. **B**. Densitometry of the pro- and anti- apoptotic proteins blots. GAPDH antibody was used as internal control for each sample. Data are represented as a mean ± standard error of the mean of three independent experiments and determined by the unpaired t- test (*P < 0.01; **P < 0.001).

To determine the effectiveness of RES induced apoptosis in PCa, cells were treated with a known concentration of RES, DTX, and RES+DTX for 48 h. Expression of the pro- and anti-apoptotic proteins in C4-2B cells after treatment of RES (47μM), DTX (10nM) and combination (RES+DTX, 20μM+10nM) showed that the up-regulation of pro-apoptotic genes (*BAX, BID*, and *BAK*) and down-regulation of anti-apoptotic genes (*MCL-1, BCL-2*, and *BCL-XL*), the changes in the protein expressions were normalized to GAPDH expression. The up-regulation of apoptotic marker cleaved PARP expression in the cells treated with the combination of drugs confirms the effectiveness of the RES and DTX in inducing apoptosis (Figure 2A). In DU145, cells were treated with the doses of RES (35μM), DTX (31nM) and combination (RES+DTX, 22μM+10nM) and after 48 h the cell death was monitored. The pro-apoptotic genes (*BAK, BID*, and *BAX*) were up- regulated in which *BAX* and *BID* expression were more significant in the cells treated with both the drugs. The down-regulation of the expression of the anti-apoptotic gene *MCL-1* was more prominent in the cells treated with both the drugs compared to *BCL-2* and *BCL-XL* as shown in densitometry (Figure 2B). Our results further demonstrated that the combined treatment enhanced the expression of cleaved *PARP* in C42B compared to DTX or RES solely; however, no significant difference was found in DU145 for cleaved *PARP* expression when treated with DTX alone or in combination with RES. These results indicate that resveratrol induces the pathway of apoptosis thus facilitating cell death.

### Resveratrol in combination with docetaxel induces the apoptotic and suppresses the anti-apoptotic markers at the mRNA level in PCa cells

**Figure 3:**
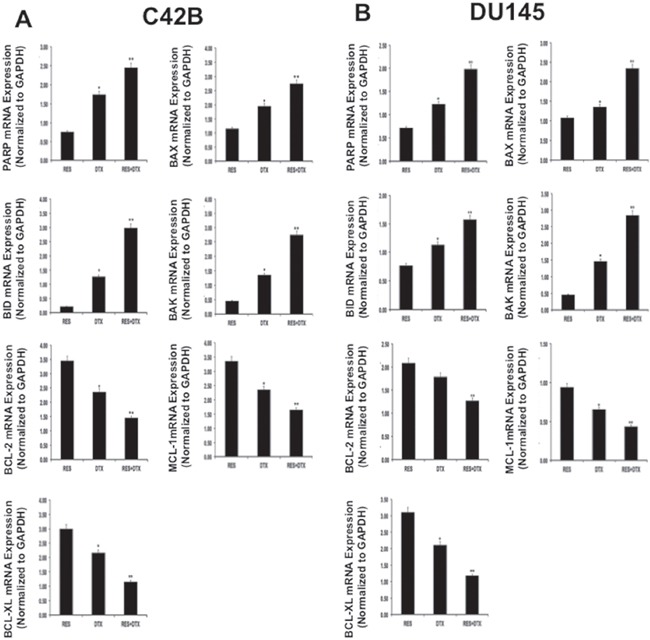
Validation of gene expression for pro-apoptotic, PARP and anti-apoptotic markers in PCa cells A C4-2B and B. DU145 by qRT-PCR. PCa cells were treated with RES, DTX and combination (RES+DTX) for 48 h. Quantitative RT-PCR analysis showed fold change expression relative to control cells for pro-apoptotic markers (BAX, BID, and BAK), PARP and anti-apoptotic markers (BCL-2, BCL-XL, and MCL-1). Data were normalized to 18S expression, and the experiments were repeated three times. Data are presented as the Mean±SEM, and asterisks indicate significance determined by student *t*-test (*P < 0.01; **P < 0.001).

To validate the previous finding on apoptosis, we further confirmed the expression of the pro-apoptotic and anti-apoptotic genes at the mRNA level after treatment. PCa cells were treated with RES, DTX and a combination of (RES+DTX) for 48 h; Quantitative RT-PCR analysis shows the mRNA expression levels of pro-apoptotic (BAX, BID, and BAK) and anti-apoptotic (BCL-2, BCL-XL, and MCL-1) markers in C4-2B and DU145 cells (Figure 3A and 3B). The expressions of MCL-1, BCL-XL and BCl-2 were significantly down-regulated in both androgen-independent DU145 and C4-2B PCa cells. The pro- apoptotic markers (BAX, BAK, and BID) and PARP were shown to be significantly up-regulated in cells treated with the combination of drugs after 48 h of treatment compared to control. This observation showed that RES enhances the docetaxel-mediated apoptosis in both cell lines.

### Resveratrol-induced cell cycle distribution in C4-2B and DU145 cells

**Figure 4:**
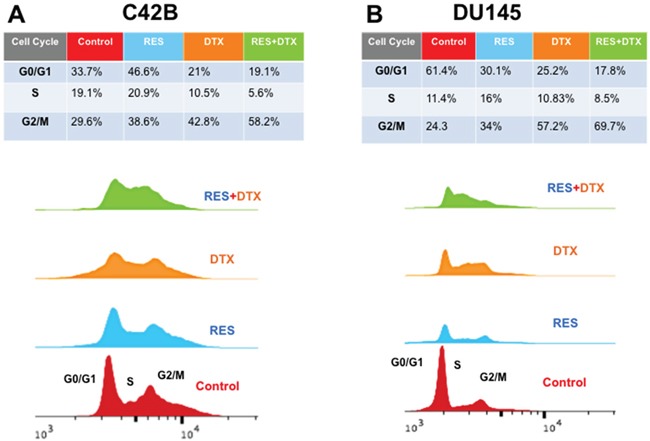
Flow cytometry analysis for cell cycle DNA content in G0/G1, S, G2/M phase after treatment of RES, DTX and RES+DTX at 48h in C4-2B and DU145 PCa cells **A**. C4-2B cells were treated with RES, DTX and the combination of RES+DTX and after 48h cells were stained with propidium iodide for cell cycle analysis and detected by flow cytometry. Percentage of cells was calculated by using FlowJo software, and a maximum number of cells was found to be arrested in the G_2_/M phase when treated with the combination of RES+DTX compared to RES or DTX alone. **B**. DU145 cells were treated with RES, DTX and the combination (RES+DTX) and after 48 h treatment, cells were processed as described earlier and cell cycle analysis were performed by Flow cytometry.

In order to examine the distribution of cell cycle progression, we confirmed the effect of RES in various cell cycle phases using Flow cytometry. RES blocked the cell cycle progression at G_1_/S or G_2_/M phase in a different way in C4-2B and DU145 cells. We calculated the area under the peaks to determine the phase within the cell cycle, using Flow Jo software v10.2. The treatment of C4-2B cells with RES (47μM) for 48h showed that the distributions of cells in G_0_/G_1_, S and G_2_/M phases were 46.6%, 20.9 % and 38.6%. However, with DTX alone (10 nM), the distributions were 21%, 10.5% and 42.8%, respectively. The combined treatment with RES+DTX (20μM+10nM) resulted in a marked increase in the percentage of cells blocked at G_2_/M phase (58.2%) and significantly reduced the percentage of cells at G_0_/G_1_ (19.1%) and S (5.6%) phases (Figure 4A). Similarly, DU145 cells treated with 35μM (RES), 31nM (DTX), 22μM+10nM (RES+DTX) for 48h showed the increased percentage of cells in G_2_/M phase (69.7%) with concomitant reduction of cells in S (8.5%) and G_0_/G_1_ (17.8%) phases (Figure 4B). These results suggest that the combination of resveratrol and docetaxel causes the highest number of cells arrested at G_2_/M phase compared to resveratrol and docetaxel alone in both PCa cell lines.

### Resveratrol modulates the expression of cell cycle inhibitor p21^WAF1/CIP1^, p27^KIP1^ and Cyclin kinases in PCa cells

**Figure 5:**
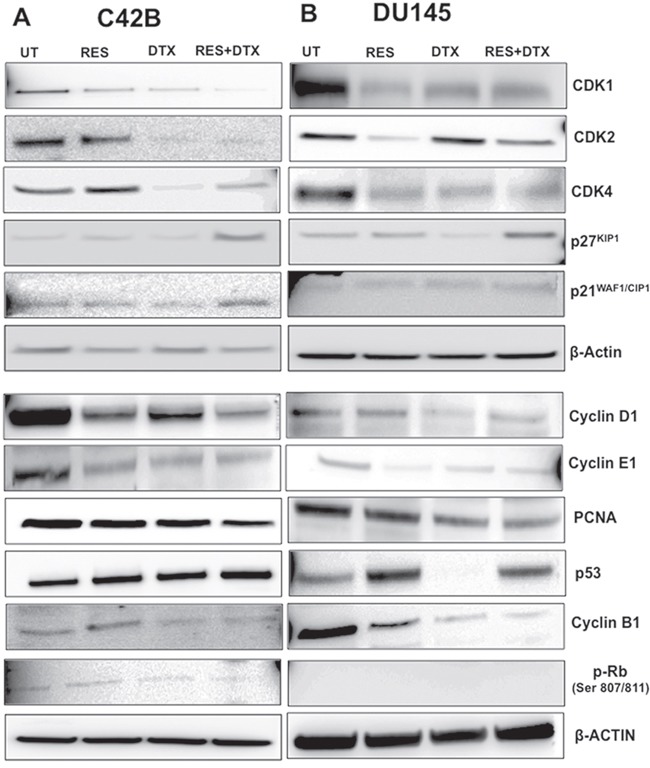
Dose-response effects of RES, DTX, and combination of RES+DTX treatment for cell cycle regulation of PCa cells 3x10^6^ cells were treated with the drugs in time and dosage- dependent manner for cell cycle regulation analysis. **A**. C4-2B cells treated by the combination of drugs [RES+DTX (20μM, 10nM)] showed upregulation of the expression of p53, cell cycle inhibitors (p21^WAF1/CIP1^and p27^KIP1^) and downregulation of p-RB, CDK/cyclin complexes compared to RES (47μM) or DTX (10nM) alone at 48 h treatment. **B**. For DU145, p53 has less expression when treated with DTX (31nM) compared to RES (35μM) and combination (RES+DTX, 22μM+ 10nM). Other cell cycles regulatory protein cyclins (D1, E, and B1) CDKs, showed the effectiveness of RES+DTX in DU145. β-Actin antibody was used as a loading control for all the samples. Data are representative of three independent experiments.

To investigate the effect of combination (RES+DTX) on cell cycle progression, we performed the western blot analysis and examined the synergistic effect on G_0_/G_1_, S and G_2_/M phase within the cell cycle in C4-2B and DU145 cells. The changes in the protein expression of cell cycle regulators, such as p21^WAF1/CIP1^, p27^KIP1^ (inhibitors of CDK family), p53, cyclin D1, CDK4 and cyclin E1, were validated, which are involved in G_1_/S transition (Figure 5A). RES activated the p53 expression and induced the increased expression of protein p21^WAF1/CIP1^and p27^KIP1^ in C4-2B cells, which are the inhibitors of CDK/cyclin complex and mainly regulate the G_1_/S transition. The enhanced expression of inhibitors p21^WAF1/CIP1^and p27^KIP1^ in cells treated with the combination of drugs decreased the expression of PCNA (proliferating cell nuclear antigen) to stop the further progression of DNA replication and cell cycle. Similarly, RES in combination with DTX inhibit the expression of cyclin D1, CDK4, cyclin E1 and induce the hypo-phosphorylation of Rb protein to inhibit the transition of cells towards G_1_/S phase. Furthermore, the combination of drugs induced a significant decrease in expression of cyclin B1 at 48h, which are involved in the G_2_/M transition.

We further examined the antitumor effect of RES in combination with DTX on cell cycle regulatory protein in DU145 cells after 48h of treatment (Figure 5B). Similar to C4-2B cells, RES enhanced the expression of p53, which induces the increased expression of p21^WAF1/CIP1^and p27^KIP1^ in combination with DTX (22μM+10nM) compared to DTX (31nM) and RES (35μM) only, which further inhibit the CDK4/cyclin D1 complex by down-regulation of the protein expression. Unlike C4-2B cells, in DU145 no significant effect on expression of CDK1, CDK2 and cyclin E1 was observed when treated with the combination of drugs compared to RES alone. Moreover, pRb was nonfunctional in the treatment groups, suggesting that inhibition of cell growth may be independent of pRb, and p53, which was also not expressed in DTX treated DU145 cells. However, the pronounced reduction of anti-proliferation marker PCNA was observed in cells treated with both the drugs. The significant reduction of cyclin B1 in combination showed the highest number of cells arrested at G_2_/M phases in DU145 cells. Together, these results of C4-2B and DU145 cells suggest that the inhibition of the cell cycle regulator by the synergistic effect of resveratrol-docetaxel combination could be one of the possible molecular events associated with the G_2_/M arrest, which leads to inhibition and cell death.

## DISCUSSION

Docetaxel is the most widely used chemotherapeutic agent to target metastatic PCa, but prolonged use of docetaxel results in drug resistance and toxicity in cancer patients. Using the natural compound in the combination therapy is a popular approach to combat the negative aspects of using a chemotherapeutic agent alone. Previous studies showed that resveratrol has anti-proliferative and pro-apoptotic effects on prostate, breast, colon cancers and other malignancies [5, 9, 22, 23]. The current study demonstrated that the combined treatment of resveratrol and docetaxel targeted the cell cycle regulatory molecules, thus blocking proliferation and inducing apoptosis, which, in turn, suppressed the growth and survival of PCa.

Our result corroborates the findings of other reports based on RES-mediated apoptosis of PCa cells. Here, we investigated the synergism between RES and DTX in enhancing the activation of the *p53* pathways, which has been shown to play a central role in the induction of apoptosis and disruption of DNA replication, thus mediating the cell cycle arrest. Our finding showed that combined drug treatment (RES+DTX) resulted in an elevated level of p53 in C4-2B cells. Upon activation of the p53 pathways, the pro-apoptotic proteins (BAX, BID, and BAK) were up-regulated and anti-apoptotic proteins (BCL-2, MCL-1, and BCL-XL) were down-regulated in C4-2B. However, in DU145 cells, the effect of combined treatment was not reflected in the p53 activation, although there was a significant increase in pro-apoptotic protein BAX and profound decrease in anti-apoptotic protein BCL-XL. The combination index (CI), which is the standard measure of the combined effect of the drugs (RES+DTX) as well supported the findings of the significantly lower synergistic effect in DU145 cells. This is also evident from the previous studies where DU145 cell line was more sensitive to docetaxel alone but has greater cell survival when two drugs were combined [24]. Moreover, the increased level of PARP expression further confirmed the effectiveness of combined drug (RES+DTX) treatment in both cell lines in inducing apoptosis, which was being supported by existing studies [25]. The expression of the proliferating marker PCNA (Acts as a scaffold to recruit protein involved in DNA replication and repair) for tumor development was also significantly down regulated in the cells treated with both RES and DTX. The down-regulation of PCNA and up-regulation of PARP cleavage further substantiates the synergistic effect of resveratrol and docetaxel on promoting apoptosis in PCa. The synergistic effects of RES and DTX on stimulating apoptosis in C4-2B and DU145 were further justified by the quantitative RT-PCR analysis.

**Figure 6:**
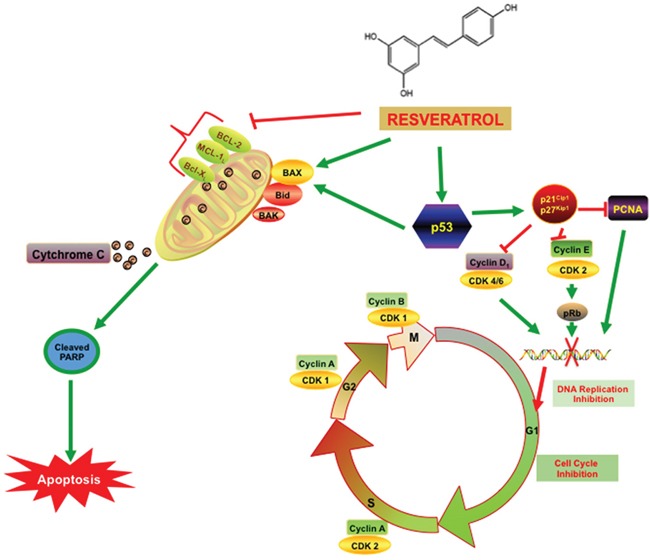
Resveratrol inhibits PCa cell proliferation via modulating molecular pathways involved in cell cycle progression and apoptosis As illustrated in the figure, cell cycle has four distinct phases: G_1_ or gap 1(cells grow and prepare to synthesize), S or synthesis (DNA Synthesizes), G_2_ or second gap (cells ready to divide) and M or mitosis phase (cell division occur). The cell cycle progression is controlled by a series of signaling cascade by which cell replicates its DNA thus allowing cell division and growth. The expression of the key regulatory gene p53 was elevated with the resveratrol treatment and also with the combination of RES+ DTX at the 48h time point. P53 acts as a transcription factor by binding to specific DNA sequences and induce the increased expression of pro-apoptotic gene BAX and cell cycle inhibitors p21^WAF1/CIP1^and p27^KIP1^. The up-regulation of BAX and down-regulation of the anti-apoptotic (BCL-2 family) gene causes the release of cytochrome C from mitochondria into the cytosol and activates caspase cascade family protein cleaved PARP that induced the apoptosis. Moreover, the p21^WAF1/CIP1^and p27^KIP1^ binds to cyclin D, E /CDK complex and inhibits kinase activity, and blocks the G_1_/S transition of the cells. The treatment with RES arrests the transition of cells at G_2_/M checkpoint by altering the CDK1/cyclin B complex formation. Moreover, the elevated level of p21^WAF1/CIP1^and p27^KIP1^ inhibits cell cycle progression through the interaction with PCNA and stops the further DNA replication.

The cell cycle progression is controlled by a series of signaling cascades by which cell replicates its DNA, divide and proliferate. Three checkpoints G_1_, S and G_2_/M control the DNA replication and cell death in cancer cells, which are controlled by *p53*, the master regulator as well as other positive (CDKs and cyclins) and negative (p21^WAF1/CIP1^, p27^KIP1^) regulators of the cell cycle (Figure 6). Up-regulation of p21^WAF1/CIP1^, p27^KIP1^, and down-regulation of cyclin D1/CDK complexes inhibit the progression through the cell cycle from G_1_ to S phase resulting in cell cycle arrest. Earlier studies demonstrated that RES up-regulates the expression of *p53* in both AD and AI cell line, which induces the G_1_/S growth arrest by the up-regulation of p21^WAF1/CIP1^, p27^KIP1^, and down-regulation of cyclin D1/CDK complexes [26–28]. In order to evaluate the potential role of RES in enhancing the effect of DTX in cell cycle arrest while used in combination, the expressions of potential key regulator proteins involved in cell cycle progression were tested [23, 29]. Our data suggested that combined treatment of the C4-2B cell line with RES and DTX indicates that the up-regulation of p53 expression stimulates the expression of p21^WAF1/CIP1^ and p27^KIP1^ and suppresses the expression of cyclin D1/CDK4 complex thus blocks the progression of cells from G_1_ to S phase. On the other hand, the E2F is the family of transcription factors that are controlled by the retinoblastoma protein pRb, and also regulates the transition of cells from G_1_ to S phase. Previous studies suggested that the hypo-phosphorylated pRB suppresses the growth and finally blocks the progression of the cells from G_1_ to S phase, but in the hyper-phosphorylation state, it promotes the progression of malignant cell in the cell cycle [30]. During G1 phase, cyclin-dependent kinases (CDKs) phosphorylate pRb with the of releases of E2F transcription factor for facilitating the genes for cell-cycle progression. The Current study revealed that the expression of the cyclin E1and CDK2 was significantly inhibited in cells treated with a combination of drugs compared to single drug alone. The down-regulation of cyclin E1/CDK2 correlates with the blocking of the cell at G_1_/S by inhibition of pRb. Similar studies have reported that cyclin E expression was increased in several malignancies such as leukemia, breast, prostate, etc. [31–33] and down-regulation of cyclin E decreased the proliferation rate of PCa cells. Further investigation on cell cycle arrest in G_2_/M phase, showed that the combination of RES with DTX treatment promotes a marked increase in the number of cells in mitosis phase, and the effect was associated with the down-regulation of cyclin B1 and CDK1 expression. Previous reports also legitimize our findings that the arrest of G_2_/M checkpoint transition is regulated by cyclin complexes and inhibitory phosphorylation of cdc-family protein [34–36].

However, RES was shown to be more effective in inducing apoptosis via upregulation of p53 compared to DTX or DTX+RES treated group in DU145. The synergistic effect of RES+DTX was also more profound in C4-2B compared to DU145 cells. Previous studies authenticated our findings, which showed that the upregulation and phosphorylation of p53 are closely associated with the docetaxel sensitivity in DU145. Although RES enhanced the effect of DTX in C4-2B in inducing apoptosis via upregulation of p53, the effect was not prominent in DU145 due to its lesser sensitivity to DTX. Additionally, the apoptosis pathway is independent of p53, which is in accordance with the previous studies [12].

The expression of p21^WAF1/CIP1^ and p27^KIP1^ was upregulated in DU145 cells with combined drug treatment. However, no significant effect on the expression of CDK1, CDK2 and cyclin E1 was observed in cells treated with RES+DTX compared to RES alone. The lack of pRB expression in DU145 cells indicates that the arrest in cell cycle checkpoint follows a different mechanism independent of pRb. Similar studies validated our findings, which showed that DU145 lacking Rb (mutated), experiences G_2_/M checkpoint arrest in presence of 17allylamino-17-demethoxygeldanamycin (17-AAG) [37]. It is further evident that p21 plays an important role in apoptosis by inducing the pro-apoptotic protein (BAX) and inhibiting anti-apoptotic protein (BCL-2) and is independent of p53 expression [18, 38, 39]. The present study also reveals that the combined treatment of RES+DTX down-regulates the expression of cyclin B1 and CDK1 in DU145 cells, which established the findings from the cell cycle study that promotes the accumulation of cells in G_2_/ M phase, which is associated with increase or decrease in the cell population in G_0_/G_1_ and S phases. Our findings in DU145 cells provide the relevance for using the combination of drugs in cell cycle arrest at G_2_/M phases, which could be the effective target for controlling the growth and proliferation of PCa cells. Likewise, another study on DU145 cells reveals that 88% cells were arrested at G_2_/M phase when treated by the combination of silibin and doxorubicin for 48 h [36]. Overall our results suggest that the combined treatment was more compelling in cell cycle arrest at G_2/_M phase in both C4-2B cells and DU145 cells compared to the resveratrol-treated group.

Finally, the present study demonstrates that RES in combination with DTX deciphered apoptosis and cell cycle arrest in PCa cell line C4-2B and DU145, although the effect was less profound in DU145. Moreover, up-regulation of apoptotic and down-regulation of the anti-apoptotic marker at protein and mRNA expression level further confirmed the effectiveness of resveratrol while used in combination with docetaxel. The higher expression of cell cycle inhibitors p21^WAF1/CIP1^, p27^KIP1^ and lower expression of cyclin-dependent kinases CDK1, CDK4, cyclin E1 and cyclin B1 in PCa cells also proves the combinatorial effect of RES and DTX, which stop the transition of cells from G_1_/S and G_2_/M phase. In conclusion, our study demonstrates the therapeutic efficacy of resveratrol in combination with low dosages of docetaxel could lead to improving the clinical outcomes of prostate cancer treatment in clinical setting.

## MATERIALS AND METHODS

### Cell lines and cell culture

Human Prostate cancer cell lines DU145 and C4-2B were cultured in RPMI-1640 medium supplemented with 10% Fetal Bovine Serum (FBS), 100 μg/ml of streptomycin, and 100 U/ml of penicillin, nonessential amino acid, HEPES, 2mM L-Glutamine and 0.025ug/ml Amphotericin-B (Fisher scientific, Pittsburgh, PA). All the Cell lines were maintained in humidified incubator containing 5% CO2 at 37°C.

### Cytotoxicity (MTT) assay

Cell proliferation assays were estimated by the MTT (3-(4,5- dimethylthiazol-2yl) 2,5-diphenyltetrazolium bromide) (Sigma, St. Louis, MO). Growing cells were trypsinized and collected using 0.25% Trypsin-EDTA (Fisher scientific, Pittsburgh, PA) and seeded in 96-well plates at a cell density of 10,000 cells /well. Cells were treated with docetaxel (5, 10, 50 nM), resveratrol (5, 10, 15, 20, 25, 50 μM) (Sigma, St. Louis, MO) and combination of drugs each well per concentration, and incubated for 24, 48 and 72 h at 37°C and 5% CO_2_ incubator. MTT (5 mg/ml) was added to each well and incubated for 2-3 h, the purple formazan crystal was dissolved in 100μL dimethyl sulfoxide (DMSO) (Sigma), and the absorbance was measured at 570 nm using a spectrophotometer (Spectramax M5, Molecular devices, Sunnyvale, CA). The IC_50_ (half maximum inhibitory concentration) value was calculated for both cell lines. After determining the IC_50_ values for RES and DTX for each C4-2B and DU145 cell line, a combination of RES+DTX were evaluated for synergy, additives, and antagonism, using combination index (CI) method introduced by Chou [40]. The combination index (CI) method was based on the non-exclusive model and following equation was used for the calculation: CI= RES/(RES)_x_ + DTX/(DTX)_x_ + (RES)(DTX)/(RES)_x_(DTX)_x_. To determine the effect of the drug combination, CI values were tested for synergy (CI<0.9), additive (0.9<CI<1.1) and antagonism (CI>1.1) respectively. The combination index experiment was repeated in triplicate at each drug concentration level, and the relative growth rates calculated in comparison with PCa cells in the absence of any cytotoxic drugs.

### Immunofluorescence assay

Twenty-thousand (20,000) cells were seeded in per well of a 12 well plate overnight and then treated with known concentration of RES, DTX and RES+DTX combination for 48h at 37°C and 5% CO_2_ incubator. After treatment cells were washed with PBS and incubated with 2drops/ml of cell viability imaging kit (Thermo Scientific, USA) for 30 minutes at 37°C. Images were captured by a fluorescent microscope with DAPI and FITC/GFP filter using EVOS FL microscope (Thermo Scientific, USA).

### Apoptosis assay

PCa cells were grown and treated with resveratrol, docetaxel and combination with the drugs for 48 h, after treatment cells were trypsinized with 0.25% trypsin, harvested and counted using hemocytometer (Countess II FL, Life Technology). The apoptosis was detected using FITC annexin V apoptosis detection kit with PI (Biolegend, San Diego, CA). About 1x10^6^ cells (DU145, C4-2B) were washed three times with phosphate-buffered saline (PBS) supplemented with 2% Fetal Bovine Serum (FBS) (Fisher scientific, Pittsburgh, PA), and then resuspended cells in Annexin V Binding Buffer at a concentration of 0.25-1.0 x 107 cells/ml. 100 μl of cell suspension was transferred in a 5 ml test tube followed by addition of 5μl of FITC Annexin V and 10μl of Propidium Iodide Solution. Cells were vortexed gently and incubated for 15 min at room temperature (25°C) in the dark then 400 μl of Annexin V Binding Buffer was added to each tube. 50,000 cells were analyzed by Flow cytometer using guava easy Cyte HT (EMD Millipore, Billarica, MA) with proper machine settings.

### Immunoblotting

Immunoblots analysis was conducted on total untreated cell lysates or lysates of DU145 and C4-2B cell lines treated with known concentration of drugs (resveratrol, docetaxel, and combination of resveratrol and docetaxel) for 48h, Protein concentrations of the cell lysates were determined by bicinchoninic acid (BCA) protein assay kit (Thermo Scientific, Rockford, IL). The equal amounts (30 μg) of protein from the cell lysates were denatured by boiling in Laemmli buffer for 5 minutes, resolved by 4-12% gradient sodium dodecyl sulfate-polyacrylamide gel electrophoresis (SDS-PAGE), and transferred to nitrocellulose membranes using iBlot dry blotting system (Thermo Scientific, Rockford, IL). The membranes were blocked in TBS (20 mM TRIS-HCl pH 7.6, 150 mM NaCl) (Fisher scientific, Pittsburgh, PA) containing 0.1% Tween 20 and 5% non-fat dry milk (Biorad, USA) for one hour at room temperature. Primary antibodies against p53, PCNA, pro-apoptotic (BAX, BAK, BID), anti-apoptotic (BCL-2, BCL-Xl, MCL-1), cleaved PARP, and cell cycle regulators (p21^WAF1/CIP1^, p27^KIP1^, p-Rb, p53, cyclin D1, cyclin B, cyclin E, CDK1, CDK2, and CDK4) purchased from Cell Signaling Technology (MA, USA) and were added to the membranes at concentration (1:500 to 1:1000 dilution) and incubated overnight at 4°C in 5% non-fat milk containing TBST (Tris-Buffered Saline-Tween 20). Membranes were then washed and corresponding horseradish peroxidase (HRP)-conjugated secondary antibodies were added for one hour followed by extra washes. Immunoreactive proteins were visualized on Image Quant LAS4000 (GE Healthcare-Biosciences, Pittsburgh, PA) using chemiluminescent detection reagent (Thermo fisher Scientific, Rockford, IL). The membranes were stripped, blocked in 5% non-fat milk for one hour, and reprobed with glyceraldehyde 3-phosphate dehydrogenase (GAPDH; Cell signaling, USA) to ensure equal loading. The band intensities were quantified using the Image-J software (NIH).

### Cell cycle analysis

Cells were plated at a density of 1x10^6^ cells /ml in each well of six well plates followed by treatment with resveratrol, docetaxel, and the combination of the drug for 48 h. After fixing with 70% ethanol for 30 min, cells were incubated with 50 μg/ml propidium iodide (Fisher scientific, Pittsburgh, PA) and 100 μg/ml RNase (Fisher scientific, Pittsburgh, PA) at room temperature in the dark for 15 min. Cells were analyzed by Flow cytometer using Guava easyCyte HT (EMD Millipore, Billarica, MA). The particular phase of the cell cycle with DNA content in G_0_/G_1_, S and G_2_/M was estimated using FlowJo software v 10.2.

### RNA isolation, cDNA synthesis and quantitative reverse transcription polymerase chain reaction (qRT-PCR)

The cells were treated for 48 h time points and lysed with Trizol reagent (Invitrogen, Paisley, UK) followed by the standard protocol for RNA extraction. Genomic DNA contamination was removed by treating with DNase (Thermo Scientific) for 30 minutes at 37°C. RNA was precipitated and resuspended in DNase, RNase-free water and quantified at 260 nm wavelength. cDNA was synthesized using 1 μg of RNA per 20 μl reaction mixture using the reverse transcription super mix for RT-qPCR reagent (Biorad, USA) and PCR condition were chosen following Bio-Rad protocol. Primers sequences for the anti-and pro-apoptotic gene were synthesized from National Center for Biotechnology Information (NCBI) gene bank database. The following sequences of the sense and antisense primers were used respectively; for BCL-2:5′-GATAACGGAGGCTGGGATGC-3′and5′-TCACTTGTGGCCCAGATAGG-3′;BCL-XL:5′-CCTG CCTGCCTTTGCCTAA-3′and5′-TGGGCTCAACCAGT CCATTG-3; MCL-1: 5′-AAGAGGCTGGGATGGGTT TG-3′ and5′-CAGCAGCACATTCCTGATGC-3′;BAK:5′- TTTACCGCCATCAGCAACCT-3′ and 5′-ATAGGCA TTCTCTGCCGTGG-3′; BAX: 5′- AAACTGGT GCTCAAGGCCC-3′and 5′-CTTCAGTGACTCGGC CAGG-3′; BID:5′-AGCACAGTGCGGATTCTGTC-3′and5′-ACCGTTGTTGACCTCACAGT-3′PARP:5′-GC TTCAGCCTCCTTGCTACA-3′and5′-TTCGCCACTTC ATCCACTCC-3′; RT-PCR were performed using SYBR® Green PCR master mix reagents (Biorad, USA) and gene expression was analyzed by CFX-manager software (CFX96 Real-Time System; Bio-Rad), 18S primer (5′-GGCCCTGTAATTGGAATGAGTC-3′and5′-CCAAGATCCAACTACGAGCTT-3′) were used as an endogenous control, and the experiments were repeated three times.

### Statistical analysis

Statistical analysis was performed as a standard error of means (±SEM) for at least three independent experiments. The level of significance was determined by one-way ANOVA and the p-values less than 0.05 were considered as statistically significant.
